# Cheese Consumption and Risk of All-Cause Mortality: A Meta-Analysis of Prospective Studies

**DOI:** 10.3390/nu9010063

**Published:** 2017-01-13

**Authors:** Xing Tong, Guo-Chong Chen, Zheng Zhang, Yu-Lu Wei, Jia-Ying Xu, Li-Qiang Qin

**Affiliations:** 1 Department of Nutrition and Food Hygiene, School of Public Health, Soochow University, 199 Renai Road, Suzhou 215123, China; tongxing@suda.edu.cn (X.T.); lsguorong@126.com (G.-C.C.); zzhang@stu.suda.edu.cn (Z.Z.); 20164247021@stu.suda.edu.cn (Y.-L.W.); 2 Key Laboratory of Radiation Biology, School of Radiation Medicine and Protection, 199 Renai Road, Suzhou 215123, China

**Keywords:** cheese, dairy, fermented food, mortality, meta-analysis

## Abstract

The association between cheese consumption and risk for major health endpoints has been investigated in many epidemiologic studies, but findings are inconsistent. As all-cause mortality can be viewed as the final net health effect of dietary intakes, we conducted a meta-analysis to examine the long-term association of cheese consumption with all-cause mortality. Relevant studies were identified by a search of the PubMed database through May 2016. Reference lists from retrieved articles were also reviewed. Summary relative risks (RR) and 95% confidence intervals (CI) were calculated using a random-effects model. Pre-specified stratified and dose-response analyses were also performed. The final analysis included nine prospective cohort studies involving 21,365 deaths. The summary RR of all-cause mortality for the highest compared with the lowest cheese consumption was 1.02 (95% CI: 0.97, 1.06), and little evidence of heterogeneity was observed. The association between cheese consumption and risk of all-cause mortality did not significantly differ by study location, sex, age, number of events, study quality score or baseline diseases excluded. There was no dose-response relationship between cheese consumption and risk of all-cause mortality (RR per 43 g/day = 1.03, 95% CI: 0.99–1.07). No significant publication bias was observed. Our findings suggest that long-term cheese consumption was not associated with an increased risk of all-cause mortality.

## 1. Introduction

Cheese, a fermented dairy product and traditionally part of the Mediterranean diet [1], is consumed by billions of people around the world. A healthy eating pattern in 2015–2020 Dietary Guidelines for Americans includes milk, yogurt, and cheese; however, the guidelines also point out that because most cheese contains more sodium and saturated fats, increased intake of dairy products would be most beneficial if more fat-free or low-fat milk products were selected rather than cheese [2]. Until now the effects of long-term cheese consumption on human health has been investigated in many epidemiologic studies, but findings are inconsistent. The scientific evidence of previous systematic review or meta-analysis indicated that the consumption of dairy foods including cheese was inversely associated with risk of type 2 diabetes (T2DM) [3]. In addition, an up-to-date meta-analysis has suggested a nonlinear inverse association between cheese consumption and risk of cardiovascular disease (CVD) [4]. On the other hand, there is some evidence that cheese consumption may be detrimental to certain diseases. A meta-analysis of prospective studies showed that a high intake of cheese was associated with increased prostate cancer risk [5], and another meta-analysis found that consumption of dairy foods, especially of milk and cheese, was associated with increased risk of Parkinson’s disease [6].

It seems that the associations between cheese consumption and risks of multiple chronic diseases have not been totally consistent, with both positive and negative associations reported. Considering the widespread consumption of cheese, a slight reduction or increase in disease risk may represent significant public health implications. All-cause mortality is used as an indicator of safety or hazard of an intervention and can be considered as the final net influence of dietary intakes on health [7]. Furthermore, four chronic diseases—cardiovascular disease, chronic respiratory disease, diabetes mellitus, and cancer—account for over 60% of all deaths globally [8]. However, a number of prospective studies that investigated the relationship between long-term cheese consumption and risk of all-cause mortality have yielded inconsistent findings. Therefore, we conducted a meta-analysis to quantify these prospective studies.

## 2. Methods

### 2.1. Literature Search

This study was conducted using a predefined protocol and in accordance with the proposed MOOSE (Meta-Analysis of Observational Studies in Epidemiology) [9]. A literature search was performed independently by two authors (XT and G-CC) on the PubMed database up to May 2016 for prospective studies of cheese consumption and risk of all-cause mortality. The following search terms were used: (1) cheese, dairy, or fermented food; (2) mortality, or death; and (3) cohort study, prospective study, or follow-up study. In addition, reference lists of relevant articles and published systematic reviews and meta-analyses were hand searched. No language restrictions were imposed.

### 2.2. Study Selection

Studies were considered eligible if they met the following criteria: (1) the study design was a prospective study; (2) relative risks (RRs) or hazard ratios (HRs) with 95% confidence intervals (CIs) for the association between cheese consumption and all-cause mortality were reported.

### 2.3. Data Extraction and Quality Assessment

Data extraction was performed using a standardized data collection form, and the information extracted was as follows: the first author’s last name, publication year, study name, duration of follow-up, number of deaths and total participants, sex and age, quantity of intake, the most fully adjusted RRs and 95% CIs, prevalent diseases excluded at baseline, and variables adjusted for in the analysis. Data were extracted by two authors (XT and G-CC). Discrepancies were resolved by discussion and, if agreement could not be achieved, by adjudication of a third reviewer (L-QQ). 

### 2.4. Statistical Analysis

A random-effects model was used to calculate summary RRs and 95% CIs for the highest compared with the lowest category of cheese consumption. RRs and corresponding SEs were logarithmically transformed to stabilize variance and normalize the distribution [10]. For one study [7] that reported the RRs stratified by sex, results were treated as two separate reports. 

Heterogeneity in studies was tested using the *Q* test [11] at a significant level of *p* < 0.1 and the *I*^2^ statistic, a quantitative measure of inconsistency across studies. To explore potential sources of heterogeneity, subgroup and meta-regression analyses were performed according to study and population characteristics including: study location, sex, age, duration of follow-up, number of events, the study quality score and baseline diseases excluded. To test the robustness of the results, a sensitivity analysis was carried out by omitting one cohort in each turn to examine the impacts of a single study on the overall effect estimate.

In addition, we quantified the dose-response relationship of cheese consumption with risk of all-cause mortality based on the method proposed by Greenland and Longnecker [12]. To perform this analysis, we estimated the distribution of cases and person-years and the RRs with the variance estimates for at least three quantitative exposure categories. When cheese consumption was reported in servings or other units, the consumption was converted into grams per day (g/day) using 43 g as a serving size, as reported in the ‘United States Department of Agriculture Food and Nutrient Database for Dietary Studies’ [13]. We further examined a potential nonlinear relationship between cheese consumption and risk of all-cause mortality by modeling exposure levels using restricted cubic splines with three knots at fixed percentiles (10%, 50%, and 95%) of the distribution [14,15]. The *p*-value for nonlinearity was calculated by testing the null hypothesis that the coefficient of the second spline was equal to zero. Potential publication bias was assessed with Egger’s test and Begg’s test [16,17] in which the log RRs were plotted against their SEs. All analyses were performed using STATA version 11.0 (StataCorp, College Station, TX, USA). *p* values < 0.05 were considered to be statistically significant.

## 3. Results

### 3.1. Literature Search

**Figure 1:**
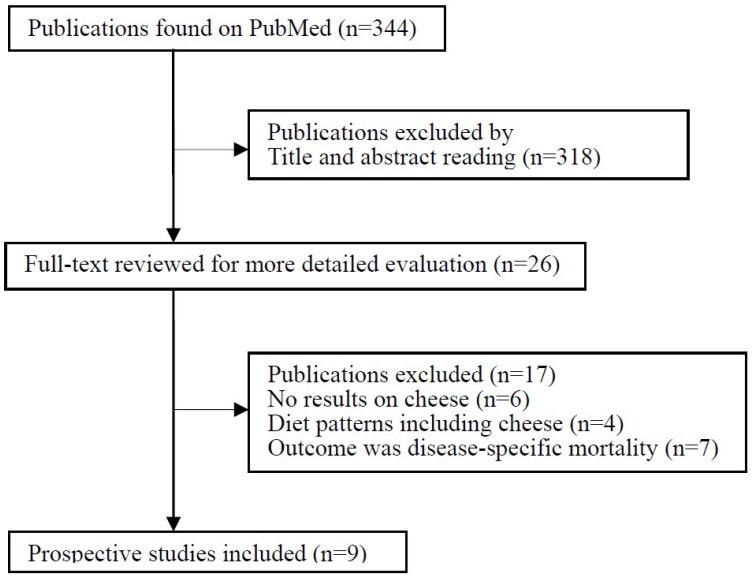
Flow chart of literature search and study selection.

The flow chart of the literature search is presented in Figure 1. The initial search of the PubMed database identified 344 records, most of which were excluded because they were not prospective studies or because the exposure or endpoint was not relevant to our analysis, leaving 26 potentially eligible papers for full-text review. Publications were further excluded mainly because the exposure of interest was dairy intake with no data on cheese consumption (*n* = 6) or was specific diet patterns including cheese (*n* = 4). Seven studies were excluded because the outcome was CVD mortality [18,19], coronary heart disease (CHD) mortality [20,21] or stroke mortality [22,23,24] without data on all-cause mortality. Finally, nine independent prospective studies were included in the meta-analysis of cheese consumption and risk of all-cause mortality [7,25,26,27,28,29,30,31,32].

### 3.2. Study Characteristics

The characteristics of the included studies are shown in Table 1. These studies were published between 1997 and 2015. Three of the studies were conducted in The Netherlands, two in the UK, two in the United States, and one each in Italy and Australia. Overall, these studies included 21,365 deaths among 177,655 participants during mean lengths of follow-up of 5 to 15 years. All studies provided adjusted risk estimates. The quality scores assigned to each study are presented in Table 2. 

### 3.3. Cheese Consumption and Risk of All-Cause Mortality

**Figure 2:**
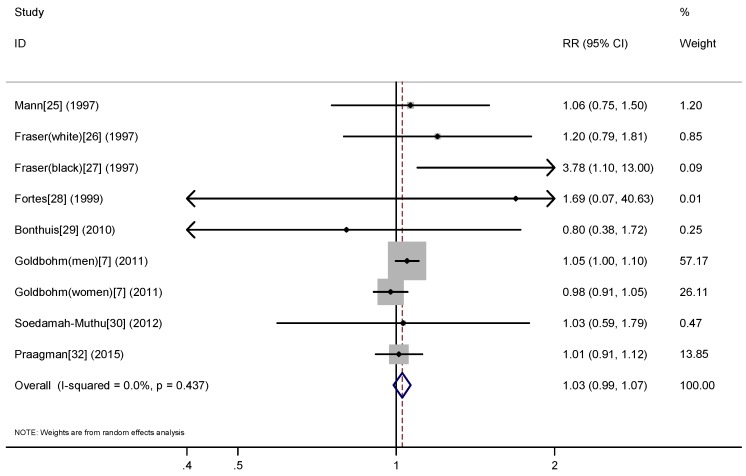
Meta-analysis of prospective studies on cheese consumption (high vs. low) and risk of all-cause mortality.

A meta-analysis of nine prospective studies yielded a summary RR of 1.02 (95% CI: 0.97–1.06) for the highest compared with the lowest cheese consumption (Figure 2), with no evidence for heterogeneity (*P*_heterogeneity_ = 0.74, *I*^2^ = 0%). There was no evidence of publication bias with Egger’s (*p* = 0.37) or with Begg’s test (*p* = 0.59).

Table 3 presents the results of subgroup analyses according to study location, sex, age, duration of follow-up, number of events, the study quality score and baseline diseases excluded. The null results were consistently observed in most of the subgroups. We found evidence of a duration-specific difference in the association (*p* = 0.09), with a marginally significant increased risk of all-cause mortality associated high cheese consumption among two studies with a duration of <10 years (RR = 1.59, 95% CI: 1.00–2.53), but not among those with a duration of ≥10 years (RR = 1.01, 95% CI: 0.96–1.06). The overall result remained stable in a sensitivity analysis in which one study at a time was omitted and the rest were analyzed, with a range from 1.00 (95% CI: 0.94–1.06) to 1.02 (95% CI: 0.97–1.08).

**Figure 3:**
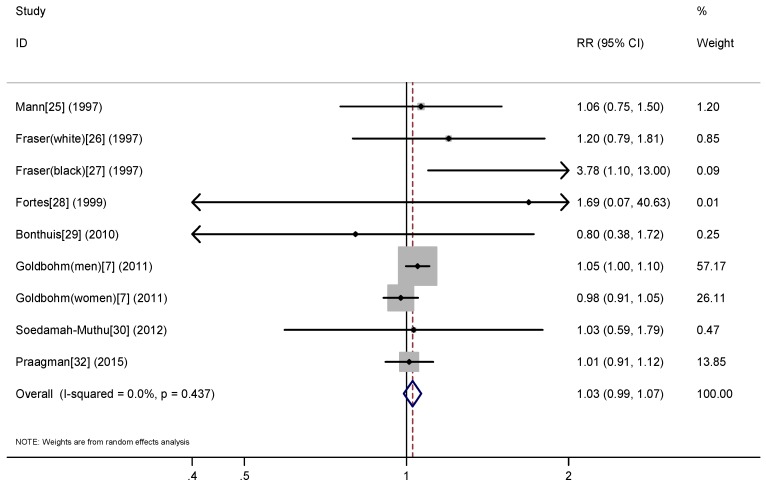
Dose-response association between cheese consumption and risk of all-cause mortality, per 50 g/day.

**Figure 4:**
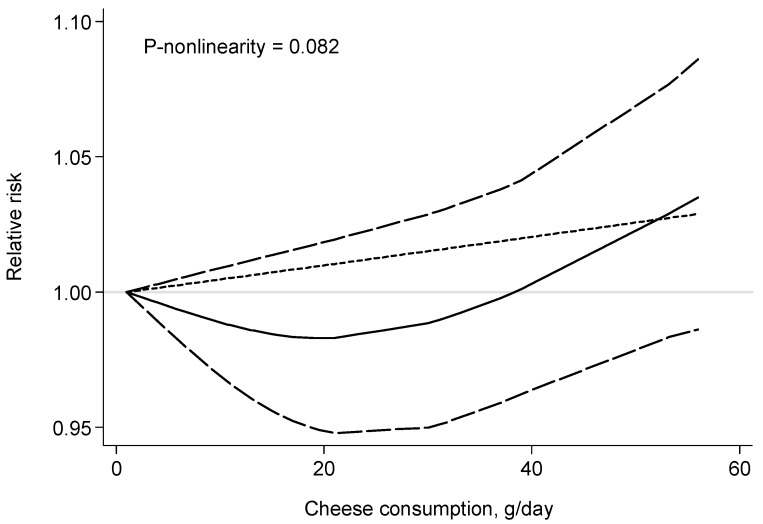
Relative risk with 95% confidence interval for the relationship between cheese consumption and risk of all-cause mortality in a restricted cubic spline random-effects meta-analysis.

Eight prospective studies [6,23,24,25,26,27,28,30] were included in the dose-response analysis of cheese consumption and risk of all-cause mortality. The summary RR (for a 43 g/day increase in the cheese consumption) was 1.03 (95% CI: 0.99–1.07) with no evidence of heterogeneity (*I*^2^ = 0%) (Figure 3). There was no evidence for a nonlinear association between cheese consumption and risk of all-cause mortality (*p* for nonlinearity = 0.082) (Figure 4).

## 4. Discussion

In this meta-analysis, we found no significant association between cheese consumption and all-cause mortality. Such a null association was consistent in stratified analysis by a number of study and population characteristics such as sex, geographical areas, baseline disease excluded, and the study quality. Dose-response analysis also found no significant association between cheese consumption and all-cause mortality. We found limited evidence for between-study heterogeneity or publication bias, and almost all studies had high quality scores (scores ≥ 8). The finding of this meta-analysis is similar with previous meta-analysis [33], which found no consistent association between milk consumption and all-cause mortality or cause-specific mortality. 

Subgroup analysis based on limited numbers of studies suggested increased risk of all-cause mortality among studies followed up for <10 years but not among those followed up for ≥10 years. The result may be explained by the fact that subjects may change their diet over time and baseline measurements would not be representative for intakes during follow-up. On the other hand, those with chronic disease such as CVD and T2DM at baseline may eat more cheese in order to have good nutrition given that cheese consumption has been reported to be inversely associated with these diseases. Unfortunately, the studies with short follow-up duration were less adjusted for dietary factors.

Although we found no association between cheese consumption and risk of all-cause mortality, there are multiple nutrients in cheese that may contribute to health benefits, such as minerals, whey protein, vitamin K_2_ and specific types of fatty acids. Evidence suggests a neutral effect of cheese consumption on blood lipids [34,35,36], which may be due to the high content of calcium in cheese. Calcium could bind with fatty acids in the intestine to form insoluble soap and lead to reduced absorption of fat, promoting a higher excretion of fecal fat [37]. Cheese is also rich in whey protein, which has been shown to reduce weight gain and blood pressure, as well as expression of inflammation and oxidative stress markers, and ultimately reduce CVD [38]. Vitamin K_2_, which is exclusively synthesized by bacteria and present in cheese, has been shown to inhibit vascular calcification and plays an important role in preventing CVD and T2DM [39,40,41].

Cheese is also one of the major food sources of saturated fat, contributing to 7.7% of total solid fat intake [42]. Saturated fat intake is reported to increase plasma levels of low-density lipoprotein-cholesterol (LDL-C) [43], a well-established risk factor for CVD [44]. The 2015–2020 Dietary Guidelines for Americans [2] pointed out that intake of saturated fats in the diet should be limited to less than 10% of energy per day and replaced with unsaturated fats, which is associated with reduced blood levels of LDL-cholesterol, and a reduced risk of CVD events and CVD-related deaths. However, a recent meta-analysis [38] reported that saturated fats were not associated with all-cause mortality, CVD, CHD, ischemic stroke, or T2DM. Furthermore, not all foods rich in saturated fats are equivalent. A randomized controlled cross-over study [45] reported that saturated fat in the form of a cheese matrix decreased postprandial inflammation compared with plant sources of saturated fat in overweight and obese individuals. Cheese is one of a number of fermented dairy products in the market. For some time now, fermented foods have been popular in the nutrition industry for their purported health benefits. In a cohort study included in the present research, total fermented food intake was not found to be associated with mortality due to all causes [32].

While dairy fat does have appreciable levels of saturated fatty acids, it is made up of nearly 400 different fatty acids, including trans-fatty acids and branched-chain fatty acids [46]. All of these unique fatty acids have a biological significance [47]. In dairy fat, conjugated linoleic acids are associated with anti-atherogenic and anti-carcinogenic effects [48,49,50], while branched-chain fatty acids are resistant to oxidation [51]. Besides the levels of saturated fats, the levels of sodium in most cheeses should be considered. A meta-analysis with 23 cohort studies and two follow-up studies of RCTs showed both low sodium intakes and high sodium intakes are associated with increased all-cause mortality [52].

These findings suggest a complexity of components in cheese. Therefore, when providing dietary recommendations on high-fat dairy or cheese, overall health effects would be more informative when comparing effects of specific components in these foods.

Our study has several strengths. The present meta-analysis is based on prospective studies, so we have effectively avoided recall and selection bias. It also included multiple country-specific studies, and the total numbers of nation-specific participants and events appear reasonably robust. In addition, we conducted a further dose-response analysis to include data across different categories of exposure by converting cheese consumption from servings into a visualized unit (g/day), and therefore largely strengthened the statistical power of the analysis. Furthermore, there was little evidence of heterogeneity in the analysis. We also noticed the individual study whose characteristics differ from others, i.e., Fraser’s study, which enrolled Seventh-Day Adventists [26,27]. Because of the unique lifestyle characteristics in this population, no dietary factors were adjusted for in the cohort. In Forbes’ study, the subjects were followed up for only five years, which is the shortest among all studies [28]. Because the subjects were elderly with a mean age of 80 years old, the overall five-year survival was assessed. On the other hand, the largest [7] or lengthiest [32] studies also provided no evidence that cheese consumption is associated with mortality due to all causes, which is highly consistent with the summary RR of the meta-analysis. Sensitivity analysis demonstrated no impacts of any single study on the overall effect estimate.

Several limitations in this meta-analysis should be considered. First, the major potential confounders adjusted for in original studies differed and residual confounders may still exist. Second, measurement errors could affect our findings as assessment of cheese consumption largely depended on self-administered questionnaires. Third, the association with high-fat cheese or low-fat cheese was not addressed because few studies reported these findings [6]. Finally, although there was no evidence of publication bias, we cannot exclude such bias because of low statistical power due to limited number of studies.

## 5. Conclusions

In conclusion, findings of the present meta-analysis indicate that cheese consumption is not significantly associated with risk of all-cause mortality. Future large prospective studies that distinguish between high-fat and low-fat cheese are warranted.

## Figures and Tables

**Table 1:** Characteristics of included prospective studies that investigated the association of cheese consumption with risk of all-cause mortality.

Author, Year (Country)	Study Name, Duration	Subjects	Age Range (Mean/Median), Years	No. of Deaths	Reported Results	Baseline Disease Excluded	Adjustments
Mann, 1997 [25] (UK)	Oxford Vegetarian Study, 13.3 years	10,802 M/F	16–79 (34.0 in M, 33.0 in F)	383	Cheese excluding cottage: ≥5 (T3) vs. <1 (T1) ser/week RR = 1.02 (95% CI: 0.76–1.37)	Cancer, angina, hypertension, HD, stroke, and diabetes	Age, sex, smoking, and social class.
Fraser (white), 1997 [26] (USA)	Adventists Health Study, 12 years	NA (11,828 person-years)	≥85	1387	Cheese: ≥3 (T3) vs. <1 (T1) ser/week RR = 1.07 (95% CI: 0.90–1.27) (M/F) RR = 1.23 (95% CI: 0.91–1.66) (M) RR = 1.01 (95% CI: 0.82–1.24) (F)	Cancer and HD	Age and sex.
Fraser (black), 1997 [27] (USA)	Black Adventists Health Study, 8 years	1668 M/F	NA (52.0 in M, 53.4 in F)	153	Cheese: ≥3 (T3) vs. <1 (T1) ser/week RR = 1.70 (95% CI: 1.00–2.90) (M/F) RR = 1.20 (95% CI: 0.50–2.80) (M) RR = 2.20 (95% CI: 1.10–4.30) (F)	None	Age, smoking and exercise.
Fortes, 1999 [28] (Italy)	NA, 5 years	162 M/F	≥65 (80)	53	Cheese: ≥2 (High) vs. <1 (Low) ser/week RR = 1.30 (95% CI: 0.51–3.34)	Mental impairment and severe disability	Age, gender, number of years of education, BMI, cigarette smoking, cognitive status and presence of chronic disease.
Bonthuis, 2010 [29] (Australia)	NA, 14.4 years	1529 M/F	25–78 (49.8)	177	Full-fat cheese: 30 (T3) vs. 4 (T1) g/day RR = 0.91 (95% CI: 0.57–1.45)	None	Age, sex, BMI, smoking, physical activity, school leaving age, dietary supplement use, β-carotene treatment during trial, use of medications for hypertension, diabetes mellitus, cardiac disorder, use of β-adrenergic blocking agents, and intakes of energy, alcohol and calcium.
Goldbohm, 2011 [7] (The Netherlands)	The Netherlands Cohort Study, 10 years	120,852 M/F	55–69 (NA)	10,658 in M; 5478 in F	Cheese: 56 (Q5) vs. 1 (Q1) g/day RR = 1.04 (95% CI: 0.96–1.12) (M) RR = 0.98 (95% CI: 0.88–1.77) (F)	Angina, MI, and stroke	Age, BMI, smoking, physical activity, education, multivitamin use, and intakes of energy, alcohol, energy-adjusted mono- and polyunsaturated fat, vegetable, and fruit.
Soedamah-Muthu, 2012 [30] (UK)	Whitehall II study, 11.7 years	4526 M/F	NA (56)	237	Cheese: 31 (T3) vs. 6 (T1) g/day RR = 1.00 (95% CI: 0.72–1.37)	CHD	Age, sex, BMI, smoking, physical activity, ethnicity, employment grade, family history of CHD/hypertension, and intakes of energy, alcohol, fruit and vegetables, bread, meat, fish, coffee, and tea.
van Aerde, 2012 [31] (The Netherlands)	Hoorn Study, 12.4 years	1956 M/F	50–75 (61.6)	403	Cheese: per 24 g/day RR = 0.96 (95% CI: 0.84–1.09)	CVD	Age, sex, physical activity, and intakes of meat, fish, bread, vegetables, fruit, coffee, and tea.
Praagman, 2015 [32] (The Netherlands)	EPIC-Netherlands, 15 years	34,409 M/F	20–70 (43.0 in M, 51.0 in F)	2436	Cheese: 53.2 (Q4) vs. 6.6 (Q1) g/day RR = 1.00 (95% CI: 0.89–1.12)	Cancer and CVD	Age, sex, smoking, BMI, physical activity, education, hypertension at baseline, and intakes of total energy, alcohol and energy-adjusted fruit and vegetables.

BMI, body mass index; CHD, coronary heart disease; CI, confidence interval; CVD, cardiovascular disease; EPIC, European Prospective Investigation into Cancer and Nutrition; F, female; HD, heart disease; M, male; MI, myocardial infarction; NA, not available; RR, relative risk.

**Table 2:** The quality of included studies assessed by the Newcastle Ottawa Scale ^a^.

	Selection				Comparability	Outcome			Total Stars
Study	Representativeness of Exposed Cohort	Selection of the Non-Exposed Cohort	Ascertainment of Exposure	Demonstration That Outcome of Interest Was Not Present at Start of Study	Comparability of Cohorts on the Basis of the Design or Analysis	Assessment of Outcome	Was Follow-Up Long Enough for Outcomes to Occur	Adequacy of Follow-Up of Cohorts	
Mann, 1997 [25]	0	1	0	1	2	1	1	0	6
Fraser (white), 1997 [26]	0	1	0	1	0	1	1	1	5
Fraser (black), 1997 [27]	0	1	0	1	0	1	1	1	5
Fortes, 1999 [28]	0	1	1	0	2	1	1	0	6
Bonthuis, 2010 [29]	0	1	1	0	2	1	1	1	7
Goldbohm, 2011 [7]	1	1	1	1	2	1	1	1	9
Soedamah-Muthu, 2012 [30]	0	1	1	1	2	1	1	1	8
van Aerde, 2012 [31]	1	1	1	1	1	1	1	0	7
Praagman, 2015 [32]	1	1	1	1	2	1	1	1	9

^a^ A study can be awarded a maximum of one star for each numbered item within the Selection and Outcome categories and a maximum of two stars for Comparability.

**Table 3:** Subgroup meta-analysis for the association of cheese consumption with risk of all-cause mortality.

Pre-Defined Factors	Subgroups	Number of Studies	RR (95% CI)	*P*_heterogeneity_	*I*^2^ (%)	*P*_interaction_
Study location	Europe	7	1.01 (0.96–1.06)	0.94	0	0.27
	USA	2	1.26 (0.82–1.93)	0.11	0.62	
Sex	Men	3	1.05 (0.98–1.13)	0.55	0	0.581
	Women	3	1.07 (0.85–1.33)	0.07	0.62	
Mean/median baseline age	≥60 years	5	1.02 (0.92–1.12)	0.42	0	0.989
	<60 years	5	1.02 (0.96–1.07)	0.72	0	
Duration of follow-up	≥10 years	8	1.01 (0.96–1.06)	0.96	0	0.09
	<10 years	2	1.59 (1.00–2.53)	0.63	0	
Number of events	≥500	4	1.00 (0.90–1.11)	0.46	0	0.69
	<500	6	1.02 (0.97–1.08)	0.77	0	
Quality score	≥8	4	1.02 (0.96–1.07)	0.84	0	0.98
	<8	6	1.02 (0.92–1.12)	0.40	0.03	
Baseline diseases excluded: CVD	Yes	7	1.01 (0.96–1.06)	0.93	80	0.33
	No	3	1.23 (0.80–1.88)	0.22	0.34	
Cancer	Yes	3	1.02 (0.93–1.12)	0.82	0	0.89
	No	7	1.01 (0.96–1.07)	0.48	0	

CVD, cardiovascular disease; *P*_heterogeneity_ value for heterogeneity among studies; *P*_interaction_ value for heterogeneity between subgroups by meta-regression analysis.
